# Effect of Energy Under-Reporting on Secular Trends of Dietary Patterns in a Mediterranean Population

**DOI:** 10.1371/journal.pone.0127647

**Published:** 2015-05-29

**Authors:** Anna N. Funtikova, Santiago F. Gomez, Montserrat Fitó, Roberto Elosua, Alejandra A. Benítez-Arciniega, Helmut Schröder

**Affiliations:** 1 Cardiovascular Risk and Nutrition Research Group (CARIN-ULEC), IMIM (Hospital del Mar Medical Research Institute), Barcelona, Spain; 2 CIBER Epidemiology and Public Health (CIBERESP), Instituto de Salud Carlos III, Madrid, Spain; 3 PhD program “Foods and Nutrition”, University of Barcelona, Barcelona, Spain; 4 Fundación THAO, Barcelona, Spain; 5 CIBER Physiopathology of Obesity and Nutrition (CIBEROBN), Instituto de Salud Carlos III, Madrid, Spain; 6 Cardiovascular Epidemiology and Genetics (EGEC-ULEC), IMIM (Hospital del Mar Medical Research Institute), Barcelona, Spain; 7 Faculty of Medicine, Autonomous University of Mexico State, Toluca, Mexico; University of East Anglia, UNITED KINGDOM

## Abstract

**Background:**

Diet is an important factor in the prevention of chronic diseases. Analysis of secular trends of dietary patterns can be biased by energy under-reporting. Therefore, the objective of the present study was to analyse the impact of energy under-reporting on dietary patterns and secular trends in dietary patterns defined by cluster analysis.

**Design and methods:**

Two cross-sectional population-based surveys were conducted in Spain, in 2000 and 2005, with 3058 and 6352 participants, respectively, aged 25 to 74 years. Validated questionnaire was used to collect dietary data. Cluster analysis was run separately for all participants, plausible energy reporters (PER), and energy under-reporters (EUR) to define dietary patterns.

**Results:**

Three clusters, “healthy”, “mixed” and “western”, were identified for both surveys. The “mixed” cluster was the predominant cluster in both surveys. Excluding EUR reduced the proportion of the “mixed” cluster up to 6.40% in the 2000 survey; this caused secular trend increase in the prevalence of the “mixed” pattern. Cross-classification analysis of all participants and PER’ data showed substantial agreement in cluster assignments: 68.7% in 2000 and 84.4% in 2005. Excluding EUR did not cause meaningful (≥15%) changes in the “healthy” pattern. It provoked changes in consumption of some food groups in the “mixed” and “western” patterns: mainly decreases of unhealthy foods within the 2000 and increases of unhealthy foods within the 2005 surveys. Secular trend effects of EUR were similar to those within the 2005 survey. Excluding EUR reversed the direction of secular trends in consumption of several food groups in PER in the “mixed” and “western” patterns.

**Conclusions:**

EUR affected distribution of participants between dietary patterns within and between surveys, secular trends in food group consumption and amount of food consumed in all, but not in the “healthy” pattern. Our findings emphasize threats from energy under-reporting in dietary data analysis.

## Introduction

Diet is a key factor in the prevention of chronic diseases [1]. Identification and promotion of healthy diets is paramount for evaluation and planning of national dietary intervention programs. Dietary pattern analysis takes into account the entire food intake and the interactions between all consumed nutrients, and has become widespread for exploring diet-disease relationships. An example of this approach is cluster analysis, which creates easily interpretable dietary patterns that are mutually exclusive [2]. This makes cluster analysis an interesting tool to explore secular trends of dietary patterns in populations. Secular trends in dietary patterns performed with cluster analysis have been analysed mainly among adolescents [3,4]. To date, only one study has been performed in the adult population [5].

All dietary assessment methods are biased by implausibly low self-reported energy intakes, a major challenge for nutritional epidemiologists [6]. Furthermore, energy under-reporting of foods seems to be selective [7]. This in turn affects dietary pattern analysis, which is based on biased dietary assessment methods. Few studies have investigated this issue and results to date are inconsistent [8].

Although the effect of energy under-reporting on secular trends of dietary pattern is unknown, it is well known that energy under-reporters tend to report healthier food choices. Additionally, it is reasonable to assume that the proportion of energy under-reporters will differ between dietary patterns. Therefore, we hypothesized that energy under-reporting might bias the time trends of dietary patterns.The aim of the present study was to explore the impact of energy under-reporting, first, on post-hoc dietary pattern analysis and, second, on secular trends of dietary patterns obtained by cluster analysis.

## Materials and Methods

### Study design and participants

Data were obtained from two population-based cross-sectional surveys of the REGICOR study (Registre Gironí del Cor) conducted in Girona (Spain) in 2000 and 2005 [9]. The 2000 survey examined a random population-based sample of 3058 men and women aged 24 to 77 years (participation rate: 71.0%); the second survey included 6352 non-institutionalized men and women older than 33 years (participation rate: 72.0%). No participants were repeated in the second survey. The present study selected only REGICOR participants aged 35 to 74 years and excluded individuals with extreme energy intake values (defined as <800 and >4200 kcal/day for men and <600 and >3500 kcal/day for women)[10]. Based on the characteristics of participants reporting extreme energy intakes according to the criteria of Willett [10], especially age and BMI, it is reasonable to assume that these values are actual outliers. Furthermore, cluster analysis is very sensitive to outliers. Therefore, we decided to exclude extreme energy intakes to avoid biased cluster formation. The exclusion of extreme intakes reduced the number of energy over-reporters; however, other surveys reported similar or even smaller proportions of energy over-reporters to the proportion defined in our study [11–13]. In total, 7373 participants (2188 from 2000 and 5185 from 2005) remained. Exclusion criteria affected the following numbers of REGICOR 2000 and REGICOR 2005 participants: age, 518 and 665; missing values in energy intake, 1 and 28; extremely low energy intake, 112 and 30; and extremely high energy intake, 239 and 444, respectively. The project was approved by the local Ethics Committee (CEIC—PSMAR, Barcelona, Spain). All participants received the results of their examination.

### Anthropometric data

Measurements were performed by a team of trained nurses and interviewers who used the same standard methods in both surveys. Weight was measured by a calibrated precision scale and rounded up to the nearest 200 g. Height was measured in the standing position and rounded up to the nearest 0.5 cm. Weight was divided by height squared (kg/m^2^) to establish the BMI. Obesity was considered with BMI ≥ 30 kg/m^2^.

### Dietary intake data

A validated [14,15] FFQ was administered by a trained interviewer to collect food consumption data. This 165-item food list, including alcoholic and non-alcoholic beverages, asked participants for their usual intakes over the preceding year. Individuals chose from 10 frequency categories, ranging from never or less than once per month to 6 or more times per day. Medium servings were defined by natural (e.g., 1 apple, 1 slice) or household units (e.g., 1 teaspoon, 1 cup).

Overall diet quality was determined by the modified Mediterranean diet score (mMDS) [16]; the published Pearson correlation for the energy-adjusted mMDS vs. multiple recalls was 0.48 [15]. The mMDS was calculated according to sex-specific, energy-adjusted tertile distribution of food consumption in the study population. For cereals, fruits, vegetables, legumes, fish, olive oil and nuts the lowest tertile was coded as 1, medium as 2, and highest as 3. For meat (including red meat, poultry and sausages) and for dairy products the score was inverted, with the highest tertile coded as 1 and lowest as 3. Moderate red wine consumption (up to 20 g) was included as a favourable component in the Mediterranean diet score, with a score of 3. Exceeding this upper limit or reporting no red wine consumption was coded as 0. Total mMDS scores ranged from 10 to 30.

### Implausible energy-reporting

Implausible energy reporters were identified by the revised Goldberg method [17,18]. Basal metabolic rate (BMR) was calculated using the Mifflin equation [19]:

$$\text{BMR}=\left({\text{Weight}}_{\text{kg}}\times 9.99\right)+\left({\text{Height}}_{\text{cm}}\times 6.25\right)-\left({\text{Age}}_{\text{y}}\;\times 4.92\right)+5\;\left(\text{among}\;\text{men}\right)$$

$$\text{BMR}=\left({\text{Weight}}_{\text{kg}}\times 9.99\right)+\left({\text{Height}}_{\text{cm}}\times 6.25\right)-\left({\text{Age}}_{\text{y}}\;\times 4.92\right)+161\;\left(\text{among}\;\text{women}\right)$$

The index of variability (S) in components of energy balance was determined. The coefficients of variability (CV) in components of energy balance were approximate values for these CV parameters derived by pooling the means of several studies [20]. The applied values for intra-individual variations in repeated measures of energy intake (CV^2^_wEI_), BMR (CV^2^_wBMR_), and physical activity level (CV^2^_tP_), were 23%, 8.5%, and 15%, respectively [20]. The number of recording days was set to 365 because the FFQ captured one year of estimated food intake. An individual physical activity level (PAL) was calculated and categorized according to quintile distribution of self-reported leisure-time physical activity (LTPA): sedentary = 1.35 (1^st^ quintile), light = 1. 55 (2^nd^ quintile), moderately active = 1.75 (3^rd^ quintile), active = 1.85 (4^th^ quintile), and vigorous = 2.2 (5^th^ quintile). Participants with BMR above or below the upper and lower 95% confidence interval limit of 1.96 standard deviations for plausible energy intake were characterized as implausible energy reporters.

The following formula was used:

$$Cut-off=PAL\times \text{exp}\left[\pm 1.96\times \frac{\left(S/100\right)}{\sqrt{n}}\right]$$

where

$$S=\sqrt{\left[\frac{CV_{wEI}^{2}}{d}+CV_{wBMR}^{2}+CV_{tP}^{2}\right]}$$

### Dietary patterns

The K-mean cluster algorithm, a non-hierarchical cluster analysis based on Euclidean distances, was used to derive dietary patterns. All individuals were placed in groups/clusters based on highest similarity and shortest distance to the cluster centre inside of the group and highest diversity and largest distance between cluster centres outside of the group.

The 165 food items of the FFQ were combined into 48 food groups according to similarities in their nutritional content. We used two methods to define clusters, based on absolute intakes of food groups and based on percentage of energy contribution of every food group. The results of both approaches did not differ significantly (not shown), therefore, according to the initial aims of the study we preferred the method using absolute intakes of food groups. To define the best cluster solution, several runs of cluster formation were performed. Criteria for cluster solutions were nutritional meaningfulness and a reasonable sample size (every cluster contained at least 5% of the study population). The final cluster solutions contained 7 and 5 clusters for 2000 and 2005, respectively. In both surveys, 3 meaningful clusters were retained and the rest of the clusters were removed as outliers due to insufficient size. In total, 32 (1.5%) and 20 (0.4%) participants in 2000 and 2005, respectively, were removed from further analysis. The same procedure was applied for separate cluster analyses in plausible energy reporters and in energy under-reporters. Among plausible energy reporters, 3 clusters remained after reaching a 5-cluster solution in both surveys and excluding from further analysis 10 (0.6%) and 15 (0.4%) participants in 2000 and 2005, respectively. Among energy under-reporters, 3 clusters remained after 6- and 5-cluster solutions in 2000 and 2005, respectively; 13 (2.2%) and 18 (1.3%) energy under-reporting participants, respectively, were excluded from further analysis.

We also performed cluster analysis in energy over-reporters, but, due to low prevalence of these participants (4.98% in the REGICOR 2000 and 4.96% in the REGICOR 2005), the cluster solutions were inconsistent. We joined the plausible energy reporters with energy over-reporters, and it resulted in similar clusters with those defined in only plausible energy reporters group. Therefore, we decided to include the energy over-reporters in the plausible energy reporters group. With fewer than 5% energy over-reporters, hardly comparable with the total proportion of plausible reporters, the combined group will be called “plausible energy reporters”, without forgetting that it includes energy over-reporters for purposes of the cluster analysis.

We also performed cluster analysis with data pooled from both surveys. As explained above, we defined a three-cluster solution in the set of data with all participants and in the set of data with only plausible energy reporters.

### Other variables

LTPA was measured by the validated Minnesota LTPA questionnaire administered by a trained interviewer [21,22]. Reported smoking habits and demographic and socioeconomic variables were obtained from structured standard questionnaires administered by trained personnel. Participants were categorized as never-smokers and ever-smokers. Maximum education level attained was elicited and recorded for analysis as primary school versus secondary school or university.

### Statistical analysis

A univariate general linear model was used to define mean values of food consumption and other variables according to the cluster distribution. To define the p-value for linear trend, we used a univariate general linear model for continuous, logistic regression for categorical, and Kruskal-Wallis H test for non-parametric variables.

To compare characteristics of the clusters between surveys and between different categories of energy reporters, we used Student t-test for continuous, χ^2^ test for categorical, and Mann-Whitney U test for non-parametric variables.

Contingency tables were used for the cross-classification of clusters of all participants and clusters of plausible energy reporters. The proportion of subjects consistently categorized (same cluster) was calculated.

Fifteen percentage difference was considered as meaningful difference in food group consumption between different groups of participants[23].

Differences were considered significant if *p* ≤0.05. Statistical analysis was performed using SPSS version 18.0. (SPSS Inc. Chicago, Ill., USA) and R.

## Results

Three clusters were identified for each survey, according to main food consumption characteristics: “healthy”, “mixed”, and “western”. The distribution of the mMDS indicated the construct validity of these clusters. Significantly higher mMDS index scores were found in “healthy” cluster members, followed by mixed and western cluster members (Table 1). Therefore, the clusters were labelled according to the diet quality of every cluster measured by the mMDS.

**Table 1:** General characteristics of clusters in the REGICOR 2000 and 2005 surveys.^a^

Variables	REGICOR 2000				REGICOR 2005			
	Healthy	Mixed	Western	*p* ^b^	Healthy	Mixed	Western	*p* ^b^
Proportion of participants, N (*%)*								
Clusters with all participants	558 (25.9)	945 (43.8)	653 (30.3)		1276 (24.7)	2349 (45.5)	1540 (29.8)	
Energy under-reporters^c^	80 (14.3)	460 (48.7)	57 (8.73)		173 (13.6)	1071 (45.6)	108 (7.01)	
Clusters with energy plausible reporters	616 (39.0)	101 (6.40)	861 (54.6)		988 (25.9)	1806 (47.3)	1021 (26.8)	
Clusters with energy under-reporters	112 (19.1)	240 (40.9)	235 (40.0)		304 (22.7)	574 (42.9)	459 (34.3)	
Age, years								
All participants	57.3	55.3*	49.3	<0.001	58.0	56.4	49.2	<0.001
	56.4, 58.2	54.7, 56.0	48.5, 50.1		57.4, 58.6	56.0, 56.8	48.7, 49.7	
Plausible reporters	57.3	55.6	50.4*	<0.001	57.7	55.3	48.8	<0.001
	56.5, 58.1	53.5, 57.6	49.7, 51.0		57.0, 58.3	54.8, 55.7	48.1, 49.4	
Under-reporters	59.3	57.6*	52.0	<0.001	57.2	59.4	51.3	<0.001
	57.3, 61.2	56.2, 58.9	50.7, 53.3		56.0, 58.3	58.5, 60.2	50.4, 52.3	
Women, %								
All participants	65.6	55.4	26.3*	<0.001	67.7	54.3	32.9	<0.001
Plausible reporters	66.9	70.3*	34.5*	<0.001	69.5	57.6	28.6	<0.001
Under-reporters	61.6	67.1*	26.0	<0.001	58.2	57.8	24.6	<0.001
High education^d^, %								
All participants	24.7**	25.7**	38.6**	<0.001	48.8	51.9	59.5	<0.001
Plausible reporters	24.8**	21.8**	37.0**	<0.001	48.9	51.5	60.2	<0.001
Under-reporters	17.9**	22.5**	30.2**	0.008	48.0	49.1	63.6	<0.001
LTPA^e^, METs min/d								
All participants	227*	195**	203	0.001	272	218	215	<0.001
	113, 405	92.1, 336	97.0, 371		174, 451	113, 378	109, 397	
Plausible reporters	196**	173	153**	0.01	249	171	203	<0.001
	91.3, 330	84.0, 294	74.9, 293		133, 393	87.0, 292	100, 367	
Under-reporters	377	262	335	<0.001	392	287	395	<0.001
	223, 519	157, 433	191, 496		259, 603	179, 483	237, 603	
Smoking^f^, %								
All participants	12.5**	17.8**	35.8**	<0.001	38.2	50.1	61.3	<0.001
Plausible reporters	11.9**	12.9**	31.8**	<0.001	38.2	48.1	64.9	<0.001
Under-reporters	5.4**	12.9**	32.8**	<0.001	46.4	45.3	63.4	<0.001
Obesity, %								
All participants	28.7	29.6*	27.0**	0.48	28.1	25.3	18.6	<0.001
Plausible reporters	25.5	26.7	22.6*	0.20	25.5	19.6	18.9	<0.001
Under-reporters	40.2	40.4	40.0**	0.96	42.4	33.1	26.4	<0.001
Energy, kcal								
All participants	2626	1914*	2852	<0.001	2647	1975	2858	<0.001
	2585, 2667	1882, 1946	2814, 2891		2621, 2673	1956, 1994	2835, 2882	
Plausible reporters	2594**	2604**	2671**	0.008	2754	2359	2983	<0.001
	2551, 2637	2498, 2710	2634, 2706		2726, 2782	2338, 2380	2956, 3011	
Under-reporters	1835	1576	1826**	0.85	1908	1572	1933	0.32
	1763, 1906	1527, 1625	1777, 1876		1870, 1946	1544, 1600	1902, 1964	
mMDS, points								
All participants	20.8**	19.2*	18.7	<0.001	21.4	19.7	18.6	<0.001
	20.5, 21.1	19.0, 19.4	18.5, 18.9		21.2, 21.5	19.5, 19.8	18.4, 18.8	
Plausible reporters^g^	20.5**	19.2	18.6	<0.001	21.3	19.4	18.5	<0.001
	20.3, 20.8	18.6, 19.8	18.4, 18.8		21.1, 21.5	19.3, 19.6	18.3, 18.7	
Under-reporters^g^	20.5*	19.4	19.7	0.03	21.4	19.8	19.4	<0.001
	19.9, 21.1	19.0, 19.8	19.3, 20.1		21.1, 21.8	19.6, 20.1	19.1, 19.7	

LTPA, leisure-time physical activity; METs, metabolic equivalents; mMDS, modified Mediterranean diet score.

*p≤0.05

**p <0.001 for differences between the REGICOR 2000 and 2005 surveys.

^a^Values are means and 95% C.I. or percentages (if specified).

^b^Polynomial contrasts were used to obtain *p* for linear trend in normal distributed continues variables (age, energy, mMDS), Kruskal-Wallis H test was used to obtain *p* value for non-parametric variables (LTPA), χ^2^ test was used to obtain *p* for linear trend for categorical variables (women, high education, smoking, obesity).

^c^The proportion of energy under-reporters in the clusters of “all participants”.

^d^More than secondary school education.

^e^Median and 25^th^ and 75^th^ percentiles.

^f^Active smokers or ex-smokers less than 1 year.

^g^ mMDS for plausible and energy under-reporters were calculated on the base of tertile distribution of food group consumption in all participants (according to survey).

In both surveys, the “mixed” cluster was the most prevalent, followed by the “western” and “healthy” clusters (Table 1). The highest proportion of energy under-reporting was found in the “mixed” cluster and the lowest in the “western” cluster in both surveys. Excluding energy under-reporters or analysing only this subgroup produced cluster solutions similar to the original data set (Table 1). Excluding energy under-reporters strongly decreased the proportion of the “mixed” cluster. Therefore, in plausible energy reporters “western” and “healthy” clusters had higher proportion of participants and the “mixed”—lower proportion in comparison with the original data set in the REGICOR 2000 survey. This was not the case in the REGICOR 2005 survey (Table 1). Cross-classification of individuals according to the original clusters and those obtained after excluding energy under-reporters showed that 68.7% in 2000 and 84.4% in 2005 were consistently placed into the same cluster.

Age and the proportion of women decreased across clusters (from “healthy” to “western”) in both surveys (Table 1). The opposite was observed for educational level and smoking. These findings were similar for cluster solutions of plausible and energy under-reporters (Table 1). The proportion of women increased in the “mixed” cluster of plausible and energy under-reporters of the 2000 survey, and was significantly higher compared to their 2005 peers. It is important to note the considerable increase in the proportion of smokers from REGICOR 2000 to REGICOR 2005.

Obesity prevalence decreased across clusters only in the REGICOR 2005 survey (Table 1). The prevalence of obesity in nearly all clusters of plausible energy reporters decreased in comparison with all reporters in both surveys. More obese individuals were found in all clusters of energy under-reporters compared to plausible energy reporters.

Diet quality measured by the mMDS decreased across clusters in both surveys, independently of energy reporting status and it was significantly higher among members of the “mixed” cluster in 2005 and of the “western” cluster in both surveys in energy under-reporters, compared to their plausible-reporter peers (Table 1).Overall dietary pattern characteristics identified by cluster analysis in all participants, plausible energy reporters and energy under-reporters were similar for both REGICOR surveys, 2000 and 2005 (Table 2). An inverse linear trend across clusters was observed for cooked and raw vegetables, pulses, cooked potatoes, fresh fish, olive oil, citrus and other fruits, nuts and low fat dairy (Table 2). A direct linear trend was found in fried potatoes, red meat, sausages, white bread, pastry, wine, fast food, soft drinks and high fat dairy.

**Table 2:** Characteristics of food groups and nutrients according to clusters of surveys in 2000 and 2005.

Variables	Healthy		Mixed		Western		*P-*trend
	2000	2005	2000	2005	2000	2005	
Cooked vegetables, g/4.2 MJ							
All participants	61.6	56.9	37.4	37.3	27.8	26.5	<0.001^a^
	58.6, 64.7	55.3, 58.4	35.0, 39.7	36.2, 38.5	25.0, 30.7	25.1, 28.0	<0.001^b^
Plausible reporters	53.1	53.7	39.0	32.9	27.4*	25.5	<0.001^a^
	50.3,56.0	52.2,55.3	32.1,46	31.8,34.1	25.0,29.7	24.0,27.1	<0.001^b^
Under-reporters	53.7*	72.5	60.9**	45.4	32.7	30.1	<0.001^a^
	46.0,61.4	68.5,76.5	55.6,66.2	42.5,48.3	27.4,38	26.9,33.4	<0.001^b^
Raw vegetables, g/4.2 MJ							
All participants	191	179	123*	113	86.9	82.7	<0.001^a^
	182, 199	174, 184	116, 129	109, 117	79.4, 94.5	78.3, 87.1	<0.001^b^
Plausible reporters	175	178	113	102	89.5*	80.5	<0.001^a^
	168,183	173,183	94.8,131	98,106	83.4,95.6	75.5,85.5	<0.001^b^
Under-reporters	159*	201	171**	131	107*	88.2	<0.001^a^
	137,180	190,212	157,186	123,139	92.5,122	79.1,97.3	<0.001^b^
Cooked potatoes, g/4.2 MJ							
All participants	34.5	33.9	26.7	27.1	24.1*	21.8	<0.001^a^
	32.0, 36.9	32.5, 35.3	24.8, 28.5	26.1, 28.1	21.9, 26.3	20.6, 23.1	<0.001^b^
Plausible reporters	32.6	32.0	26.0	26.4	23.8*	21.5	<0.001^a^
	30.4,34.7	30.4,33.5	20.8,31.3	25.2,27.5	22.0,25.6	20.0,23.0	<0.001^b^
Under-reporters	34.7	35.1	32.7	29.5	23.7	24.9	0.004 ^a^
	28.5,40.9	32.1,38.1	28.4,36.9	27.3,31.7	19.4,28.0	22.4,27.3	<0.001 ^b^
Fried potatoes, g/4.2 MJ							
All participants	2.15	2.12	3.59*	3.02	6.63**	5.55	<0.001^a^
	1.73, 2.57	1.89, 2.34	3.27, 3.92	2.85, 3.18	6.24, 7.02	5.34, 5.75	<0.001^b^
Plausible reporters	2.36	2.1	3.83	3.2	5.79	5.93	<0.001^a^
	1.96,2.75	1.85,2.35	2.86,4.80	3.01,3.38	5.46,6.13	5.69,6.18	<0.001^b^
Under-reporters	3.39*	1.95	2.44	2.28	4.93	5.35	0.013 ^a^
	2.38,4.39	1.47,2.43	1.75,3.12	1.93,2.63	4.24,5.63	4.96,5.74	<0.001^b^
Pulses, g/4.2 MJ							
All participants	34.7	34.9	34.1**	29.4	29.8*	27.6	<0.001^a^
	32.7, 36.8	33.9, 35.9	32.5, 35.6	28.7, 30.2	27.9, 31.6	26.7, 28.5	<0.001^b^
Plausible reporters	32.8	33.6	28.4	28.0	29.3*	27.6	0.001^a^
	31.2,34.4	32.6,34.7	24.4,32.5	27.2,28.7	27.9,30.7	26.6,28.6	<0.001^b^
Under-reporters	59.5**	36.1	31.3	31.3	36.4*	32.2	<0.001^a^
	54.1,64.9	33.7,38.4	27.6,35	29.6,33.0	32.7,40.2	30.3,34.1	0.013 ^b^
Red meat, g/4.2 MJ							
All participants	29.7*	31.8	39.0	38.6	47.2	45.5	<0.001^a^
	28.0, 31.4	30.7, 32.9	37.6, 40.3	37.8, 39.5	45.6, 48.8	44.5, 46.5	<0.001^b^
Plausible reporters	30.4	31.8	34.7	37.6	45.4*	47.8	<0.001^a^
	28.9,32.0	30.6,33	30.9,38.5	36.7,38.5	44.1,46.7	46.7,49.0	<0.001^b^
Under-reporters	36.1	36.1	33.1*	37.8	48.4*	43.9	<0.001^a^
	31.7,40.5	33.5,38.6	30.1,36.1	35.9,39.6	45.4,51.4	41.8,46.0	<0.001^b^
Sausages, g/4.2 MJ							
All participants	5.92*	6.49	7.08*	7.58	10.2	10.2	<0.001^a^
	5.38, 6.45	6.13, 6.85	6.67, 7.49	7.31, 7.84	9.66, 10.7	9.88, 10.5	<0.001^b^
Plausible reporters	6.31	6.27	5.70*	7.69	9.28**	10.7	<0.001^a^
	5.79,6.84	5.88,6.66	4.41,6.99	7.40,7.98	8.84,9.73	10.4,11.1	<0.001^b^
Under-reporters	5.74**	7.88	5.24**	7.08	9.82	9.16	<0.001^a^
	4.68,6.80	7.06,8.71	4.51,5.96	6.48,7.68	9.09,10.6	8.48,9.83	<0.001^b^
Fresh fish, g/4.2 MJ							
All participants	31.2	28.9	20.5	20.2	16.4*	15.2	<0.001^a^
	29.6, 32.7	28.1, 29.7	19.3, 21.6	19.6, 20.8	15.0, 17.8	14.5, 16.0	<0.001^b^
Plausible reporters	26.9	27.8	19.8	17.9	16.5*	15.0	<0.001^a^
	25.7,28.2	27.0,28.7	16.6,23.0	17.3,18.5	15.4,17.6	14.2,15.9	<0.001^b^
Under-reporters	29.0*	34.9	27.6	24.0	20.8*	17.3	0.003 ^a^
	24.6,33.5	33.0,36.9	24.5,30.6	22.6,25.4	17.7,23.9	15.7,18.9	<0.001^b^
Rice & Pasta, g/4.2 MJ							
All participants	24.8*	27.2	28.3	28.5	24.9*	27.9	0.911 ^a^
	22.9, 26.6	26.1, 28.3	26.9, 29.7	27.6, 29.3	23.2, 26.6	26.9, 28.9	0.359 ^b^
Plausible reporters	25.5	27.5	26.8	26.6	24.8*	27.8	0.537 ^a^
	24.0,27.0	26.2,28.7	23.1,30.4	25.6,27.5	23.6,26.1	26.6,29.0	0.723 ^b^
Under-reporters	25.7	27.9	31.4	31.2	29.5	31.1	0.261 ^a^
	20.2,31.1	25.7,30.2	27.7,35.1	29.5,32.9	25.7,33.2	29.3,33.0	0.035 ^b^
White bread, g/4.2 MJ							
All participants	17.8	18.0	26.8	25.9	24.6	24.0	<0.001^a^
	16.1, 19.4	16.9, 19.0	25.5, 28.0	25.1, 26.7	23.1, 26.1	23.0, 25.0	<0.001^b^
Plausible reporters	19.9*	17.5	21.8*	27.5	25.7**	22.8	<0.001^a^
	18.4,21.3	16.3,18.6	18.3,25.3	26.6,28.3	24.6,26.9	21.7,23.9	<0.001^b^
Under-reporters	26.8**	13.3	21.9	24.5	28.2	26.5	0.615 ^a^
	22.4,31.2	10.9,15.6	18.9,24.9	22.8,26.2	25.1,31.2	24.6,28.4	<0.001^b^
Olive oil, g/4.2 MJ							
All participants	12.5	12.4	11.3**	13.2	8.56*	9.30	<0.001^a^
	11.9, 13.2	12.1, 12.8	10.8, 11.8	12.9, 13.5	7.97, 9.16	8.95, 9.65	<0.001^b^
Plausible reporters	12.7	12.2	10.5*	13.1	9.21	8.78	<0.001^a^
	12.1,13.3	11.7,12.6	9.05,11.9	12.8,13.4	8.72,9.7	8.37,9.19	<0.001^b^
Under-reporters	8.84**	13.56	15.1	13.8	8.45*	9.80	0.678 ^a^
	7.34,10.3	12.7,14.4	14.0,16.1	13.1,14.4	7.42,9.49	9.09,10.5	<0.001^b^
Pastry, g/4.2 MJ							
All participants	2.92*	2.09	4.30*	3.57	6.78	7.49	<0.001^a^
	2.24, 3.59	1.70, 2.48	3.78, 4.82	3.28, 3.85	6.15, 7.40	7.14, 7.84	<0.001^b^
Plausible reporters	3.37**	2.1	3.06*	4.30	6.40**	8.34	<0.001^a^
	2.74,4.01	1.64,2.56	1.5,4.61	3.96,4.64	5.86,6.93	7.88,8.79	<0.001^b^
Under-reporters	1.77	1.47	3.35	2.86	5.16	4.5	<0.001^a^
	0.225,3.32	0.809,2.14	2.29,4.4	2.37,3.34	4.09,6.23	3.96,5.04	<0.001^b^
Citrus fruits, g/4.2 MJ							
All participants	152**	110	85.3*	74.0	46.8*	41.0	<0.001^a^
	145, 160	106, 114	79.5, 91.1	71.0, 77.0	39.8, 53.9	37.3, 44.7	<0.001^b^
Plausible reporters	142**	115	109**	65.7	52.1**	38.8	<0.001^a^
	135,148	111,119	92.6,125	62.5,69	46.6,57.6	34.5,43.1	<0.001^b^
Under-reporters	200**	100	85.9	95.9	44.5	41.9	<0.001^a^
	181,219	90.8,109	73.2,98.6	89.4,103	31.6,57.3	34.5,49.2	<0.001^b^
Other fruits, g/4.2 MJ							
All participants	222**	151	129**	108	81.3*	74.9	<0.001^a^
	213, 231	147, 156	122, 136	105, 112	73.2, 89.5	70.6, 79.1	<0.001^b^
Plausible reporters	209**	156	139**	104	88.1**	68.8	<0.001^a^
	201,217	151,161	118,159	100,108	81.1,95.2	63.7,73.9	<0.001^b^
Under-reporters	206**	129	164*	135	84.1*	73.6	<0.001^a^
	186,226	120,139	150,177	128,142	70.3,97.8	65.6,81.6	<0.001^b^
Nuts, g/4.2 MJ							
All participants	6.42*	7.60	4.46	4.51	4.32	4.22	<0.001^a^
	5.86, 6.99	7.26, 7.94	4.02, 4.89	4.26, 4.76	3.80, 4.85	3.91, 4.54	<0.001^b^
Plausible reporters	6.05	8.18	5.01	4.63	4.50	4.08	<0.001^a^
	5.51,6.58	7.79,8.58	3.7,6.33	4.34,4.92	4.04,4.95	3.69,4.47	<0.001^b^
Under-reporters	3.82*	5.15	5.52	4.71	3.53	4.04	0.715 ^a^
	2.52,5.13	4.5,5.81	4.63,6.41	4.23,5.19	2.63,4.43	3.5,4.57	0.010 ^b^
Wine, g/4.2 MJ							
All participants	17.0	15.8	33.8*	28.3	46.6**	29.9	<0.001^a^
	13.1, 20.9	13.7, 17.9	30.8, 36.8	26.8, 29.9	43.0, 50.2	28.0, 31.8	<0.001^b^
Plausible reporters	17.3	15.2	22.5	25.6	44.7**	31.7	<0.001^a^
	13.7,20.8	13.0,17.4	13.8,31.3	23.9,27.2	41.7,47.7	29.5,33.9	<0.001^b^
Under-reporters	16.1	20.0	20.9	24.0	58.4**	41.9	<0.001^a^
	7.05,25.1	15.1,24.9	14.8,27.1	20.4,27.5	52.1,64.6	37.9,45.8	<0.001^b^
Softdrinks, g/4.2 MJ							
All participants	6.06	5.24	10.8	11.5	21.1*	28.7	<0.001^a^
	3.01, 9.11	3.07, 7.42	8.46, 13.1	9.91, 13.1	18.2, 23.9	26.7, 30.7	<0.001^b^
Plausible reporters	6.41	5.01	7.37	11.56	19.3**	33.0	<0.001^a^
	3.41,9.42	2.57,7.46	-0.05,14.8	9.75,13.4	16.7,21.8	30.6,35.4	<0.001^b^
Under-reporters	3.13	5.40	7.82	8.36	17.0*	25.2	<0.001^a^
	-2.98,9.23	0.91,9.89	3.65,12.0	5.10,11.6	12.8,21.2	21.5,28.9	<0.001^b^
High fat dairy, g/4.2 MJ							
All participants	30.5	31.6	55.0*	46.1	59.4	62.6	<0.001^a^
	24.7, 36.3	28.1, 35.1	50.6, 59.5	43.5, 48.6	54.0, 64.7	59.4, 65.7	<0.001^b^
Plausible reporters	33.8	30.0	52.4	55.4	65.5	59.3	<0.001^a^
	28.4,39.2	26.1,34	39,65.7	52.4,58.3	61.0,70.1	55.4,63.1	<0.001^b^
Under-reporters	38.1*	22.5	36.6	36.2	51.9	58.3	0.089 ^a^
	24.9,51.2	15.5,29.5	27.7,45.6	31.1,41.3	42.8,60.9	52.6,64	<0.001 ^b^
Low fat dairy, g/4.2 MJ							
All participants	104	107	81.9*	91.4	30.1*	37.7	<0.001^a^
	96.9, 112	102, 112	76.2, 87.6	88.0, 94.8	23.2, 37.0	33.4, 41.9	<0.001^b^
Plausible reporters	101	108	96.8	73.6	39.5*	33.1	<0.001^a^
	94.4,107	104,113	81.1,113	70.1,77.2	34.1,44.9	28.4,37.8	<0.001^b^
Under-reporters	69.1**	107	149	139	29.7**	46.2	0.001^a^
	50.8,87.3	96.5,118	137,162	131,147	17.1,42.3	37.3,55.1	<0.001^b^
Fast food, g/4.2 MJ							
All participants	0.13	0.1	0.30*	0.17	0.69	0.63	<0.001^a^
	-0.03,0.28	0.02, 0.18	0.19, 0.42	0.10, 0.23	0.55, 0.83	0.55, 0.70	<0.001^b^
Plausible reporters	0.11	0.09	0.12	0.16	0.57*	0.77	<0.001^a^
	-0.01,0.24	-0.01,0.19	-0.19,0.44	0.08,0.23	0.46,0.67	0.68,0.87	<0.001^b^
Under-reporters	0.01	0.08	0.37	0.08	0.53	0.25	0.099 ^a^
	-0.33,0.52	-0.02,0.17	0.08,0.66	0.007,0.15	0.24,0.82	0.17,0.33	0.008^b^
Carbohydrates, %							
All participants	45.1	42.8	41.6	40.8	39.1	40.0	<0.001^a^
	44.4, 45.7	42.4, 43.1	41.1, 42.1	40.5, 41.1	38.6, 39.7	39.7, 40.4	<0.001^b^
Plausible reporters	44.9	43.1	40.6	40.8	39.4	39.6	<0.001^a^
	44.3,45.5	42.7,43.6	39.2,42.1	40.5,41.1	38.9,39.9	39.2,40.0	<0.001^b^
Under-reporters	46.6	40.5	42.3	41.8	39.6	40.4	<0.001^a^
	45.1,48.1	39.7,41.3	41.3,43.3	41.2,42.4	38.6,40.6	39.7,41.0	0.799 ^b^
Proteins, %							
All participants	18.5	18.7	17.9	17.5	17.2	16.9	<0.001^a^
	18.3, 18.8	18.5, 18.8	17.7, 18.1	17.4, 17.6	17.0, 17.4	16.7, 17.0	<0.001^b^
Plausible reporters	18.1	18.5	17.0	16.9	17.2	16.8	<0.001^a^
	17.9,18.3	18.3,18.6	16.5,17.5	16.8,17.0	17.0,17.4	16.7,17.0	<0.001^b^
Under-reporters	19.1	20.1	18.8	18.4	18.0	17.3	0.006^a^
	18.4,19.7	19.7,20.4	18.4,19.3	18.1,18.6	17.5,18.4	17.0,17.5	<0.001^b^
Lipids, %							
All participants	37.8	40.1	40.2	41.7	41.2	42.5	<0.001^a^
	37.2, 38.4	39.7, 40.4	39.8,40.7	41.5, 42.0	40.7, 41.7	42.2, 42.8	<0.001^b^
Plausible reporters	38.3	39.9	42.9	42.5	41.5	42.5	<0.001^a^
	37.8,38.9	39.6,40.3	41.5,44.2	42.2,42.8	41,41.9	42.2,42.9	<0.001^b^
Under-reporters	35.6	40.5	39.8	40.4	39.4	41.0	<0.001^a^
	34.2,37.0	39.7,41.2	38.9,40.8	39.9,40.9	38.4,40.3	40.4,41.6	0.252 ^b^
Energy, MJ							
All participants	11.0	11.1	8.01*	8.26	11.9	12.0	<0.001^a^
	10.8, 11.2	11.0, 11.2	7.87, 8.14	8.18, 8.34	11.8,12.1	11.9, 12.1	<0.001^b^
Plausible reporters	10.9**	11.5	10.9**	9.87	11.2**	12.5	0.008 ^a^
	10.7, 11.0	11.4,11.6	10.5, 11.3	9.78, 9.96	11.0, 11.3	12.4, 12.6	<0.001^b^
Under-reporters	7.68*	7.98	6.59	6.58	7.64	8.09	0.848 ^a^
	7.38, 7.97	7.82, 8.14	6.39, 6.80	6.46, 6.69	7.43, 7.85	7.96, 8.22	0.324 ^b^

Values are means and 95% C.I. or percentages and 95% C.I.

*P* for linear trend was obtained using polynomial contrasts.

^a^p-trend for the REGICOR 2000 survey.

^b^p-trend for the REGICOR 2005 survey.

*p-value ≤0.05 and **p-value<0.001 for the comparison of the REGICOR 2000 and 2005.

### Energy under-reporting and dietary patterns within surveys

**Fig 1:**
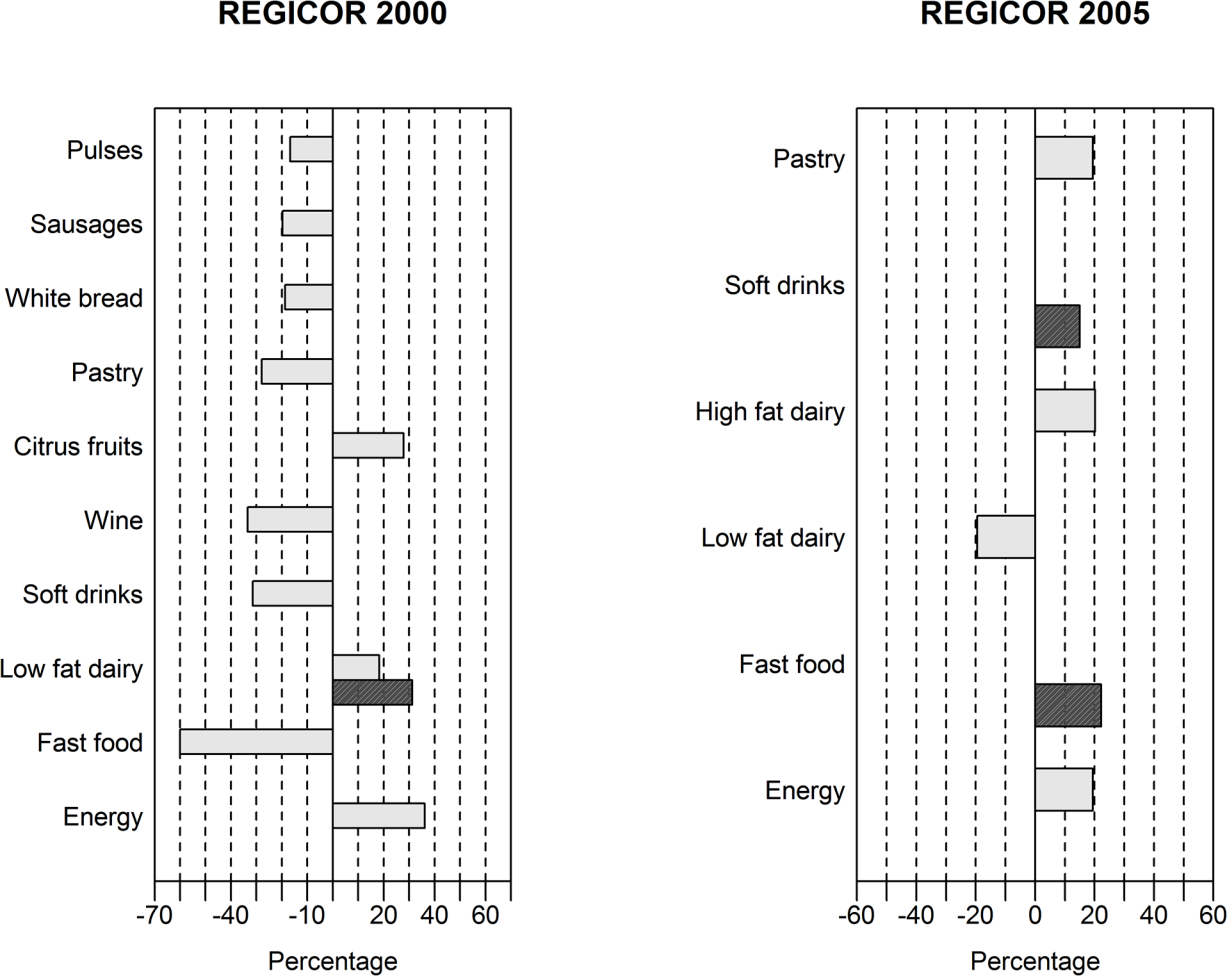
Meaningful* percentage changes in absolute intake of food group consumption in grams in plausible energy reporters after excluding energy under-reporters within 1) the REGICOR 2000 and 2) the REGICOR 2005 surveys. Only groups of food are included, which had meaningful changes. White—mixed dietary patter, black—western dietary pattern. *≥15% difference in food group consumption in plausible energy reporters compared with all reporters.

The amount of food consumption within the same survey was affected after excluding energy under-reporters from analysis (Fig 1). The “healthy” pattern did not have any meaningful changes in food consumption after exclusion of energy under-reporters, both in the REGICOR 2000 and 2005 surveys. Most of the changes occurred in the “mixed” pattern. The decreases occurred in consumption of such unhealthy food, as sausages, white bread, pastry, soft drinks and fast food, and such healthy food group, as pulses; and increases in such healthy food groups, as low fat dairy and citrus fruits, in the REGICOR 2000 survey. In the REGICOR 2005 survey the opposite was true. Energy consumption increased meaningfully only in the “mixed” pattern in both surveys (Fig 1). The “western” pattern was slightly affected by excluding energy under-reporters, only low-fat dairy in the REGICOR 2000 survey, and soft drinks and fast food in the REGICOR 2005 survey were increased.

### Energy under-reporting and secular trends, between surveys

**Fig 2:**
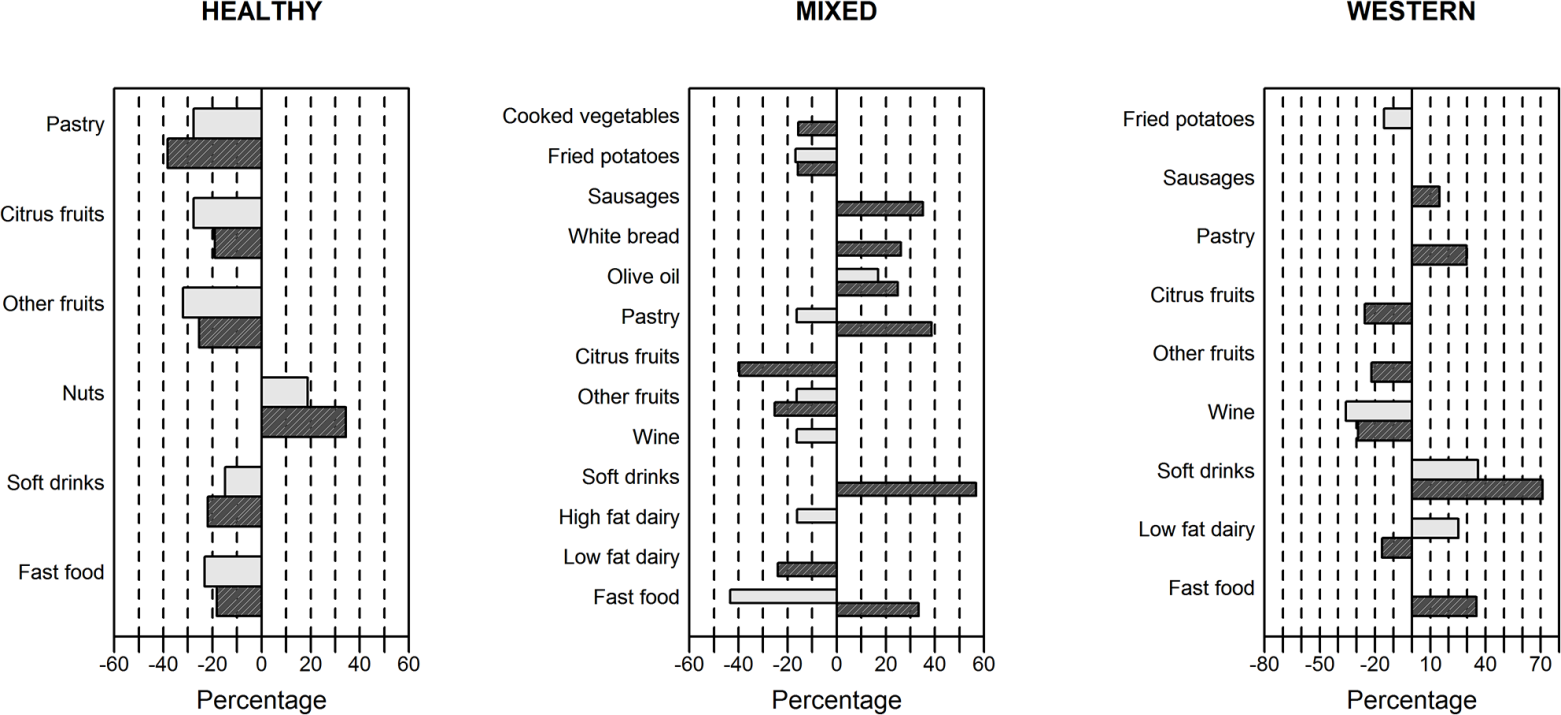
Meaningful* percentage temporal changes in absolute intake of food group consumption in grams of secular trends in all reporters and plausible energy reporters in the 1) “healthy”, 2) “mixed” and 3) “western” patterns. Only groups of food are included, which had meaningful changes. The analysis was unadjusted for population differences between the two surveys in age, sex, smoking and physical activity. The unhealthy perceived food groups go first, the healthy food groups are in continuation. A positive value means an increase from the REGICOR 2000 to the REGICOR 2005 and a negative value means a decrease from the REGICOR 2000 to the REGICOR 2005. White—all reporters, black—plausible energy reporters. *≥15% change in food group consumption compared between the REGICOR 2000 and the REGICOR 2005 surveys.

Excluding energy under-reporters had consequences for secular trends of food consumption and prevalence of participants in the same type of cluster between surveys (Fig 2). Secular trend of prevalence of the participants shifted towards higher prevalence of the “mixed” pattern with 40.9% increase in the REGICOR 2005 survey, and subsequent decreases of the “healthy” and “western” patterns, 13.1% and 27.8%, respectively.

#### Less healthy perceived food

In the “healthy” cluster, all food groups with meaningful changes of secular trends had the same direction in both all reporters and plausible energy reporters (Fig 2). Among them, pastry, soft drinks, and fast food consumption decreased. Main changes in the food consumption occurred in the “mixed” dietary pattern. The direction of meaningful secular trends changed to the opposite direction in pastry and fast food in this pattern (Fig 2). In the “mixed” cluster six food groups showed meaningful changes only in plausible energy reporters, where the unhealthy food groups, such as sausages, white bread and soft drink increased the consumption. In the same cluster high fat dairy only in all reporters and fried potatoes in both, all and plausible energy reporters decreased meaningfully (Fig 2). In the “western” cluster consumption of sausages, white bread, and pastry increased only in plausible energy reporters, and soft drinks in both all and plausible energy reporters (Fig 2).

#### Healthy perceived foods

In the “healthy” cluster all fruits consumption decreased and nuts consumption increased over time (Fig 2). Such food groups of the “mixed” clusters, as cooked vegetables, citrus fruits and low fat dairy decreased only in plausible energy reporters. However, consumption of olive oil increased in both all and plausible energy reporters (Fig 2). In the “western” pattern the direction of meaningful secular trends changed to the opposite direction in low fat dairy, it decreased in plausible energy reporters. Also, fruits and wine consumption decreased in plausible energy reporters only and in both all and plausible energy reporters, respectively (Fig 2).

#### Pooled analysis

Cluster analysis of the data pooled from both surveys gave similar cluster solutions to the clusters analysed separately in every survey (not shown). Clusters with all participants and only with plausible energy reporters had three-cluster solutions, with “healthy”, “mixed” and “western” patterns, similar to the cluster solutions with all participants and plausible energy reporters in each survey. In plausible energy reporters, we defined two sets of three-cluster solutions, which had similar clusters but different proportions. One set had similar proportions between clusters and another set had a very low proportion of participants in the “mixed” cluster (not shown).

## Discussion

Using cluster analysis of both cross-sectional surveys, REGICOR 2000 and REGICOR 2005, we identified three dietary patterns: “healthy”, “mixed” and “western”. Similar dietary patterns were defined in all participants, plausible energy reporters, and energy under-reporters. Energy underreporting affected distribution of the participants between clusters, secular trends of food consumption and amounts of food consumed in the “mixed” and “western” dietary patterns. Our study is the first to construct dietary patterns for energy under-reporters and analyse them separately from data for all participants and for plausible energy reporters.

The dietary patterns defined in our study population resembled those defined in other populations in Europe and the USA [24–34]. Most of these studies had analogues to our “healthy”, “mixed” and “western” patterns regarding sociodemographic, lifestyle and dietary characteristics. The proportion of energy under-reporters in our population (26%-27%) was comparable with some studies [35–37] but lower than others [29,32,38]. The distribution of energy under-reporters among dietary patterns also differed between studies. In several studies, a “healthy” dietary pattern had the highest proportion of energy under-reporters [32,38]. In another study, energy under-reporters among women were distributed evenly [29], but Martikainen et al. [37] reported uneven distribution. In the present study, the gender distribution between different dietary patterns was uneven in all groups of participants. Also, the “mixed” pattern had the highest proportion of energy under-reporters and the highest number of participants, similar to the “convenience” dietary pattern in men reported by Pryer et al. [29]. Therefore, the “mixed” pattern in the REGICOR 2000 survey was most affected by energy under-reporting.

### Effect of under-reporting on post-hoc dietary pattern analysis within surveys

Excluding energy under-reporters did not alter the general structure of the dietary patterns. At the same time, excluding energy under-reporters affected socio-demographic and lifestyle characteristics, food consumption, and distribution of participants between patterns. Bailey et al. [35] showed that seven of 25 food groups of dietary patterns changed consistently after excluding energy under-reporters, in the present study we found nine and five groups out of twenty with meaningful changes within the REGICOR 2000 and 2005 surveys respectively. The “healthy” pattern was not affected meaningfully by exclusion of energy under-reporters, probably, due to low prevalence of energy under-reporters in this dietary pattern and similarity of diet quality, according to the mMDS measurement between all participants and energy under-reporters of the “healthy” cluster. The strongest impact the exclusion of energy under-reporters had on the “mixed” pattern, especially in the REGICOR 2000 survey. The dramatic decrease in the proportion of participants in the “mixed” pattern of the REGICOR 2000 after exclusion of energy under-reporters (43.8% vs. 6.40%) underlined the importance of considering energy under-reporters in the analysis of nutritional surveys data and in the construction of dietary patterns. In the “western” cluster just few food groups were affected by excluding energy under-reporters. This difference in effect of energy under-reporters on the dietary patters partially could be due to different prevalence of energy under-reporters in the patterns. In a study using principal component analysis [36], nutrient intakes were slightly higher in the patterns with plausible energy reporters, but the association of nutrients with dietary patterns remained the same. In another study [32], the dietary patterns remained similar after exclusion of energy under-reporters and in one more study [37], 70% of plausible energy reporters fell into the same dietary patterns as in the analysis of all participants. This was comparable with the results obtained in the present study. It is of importance to note that the exclusion of energy under-reporters in the REGICOR 2000 caused meaningful changes both in healthy and unhealthy food groups and in different directions, with slight predominance of decreases in unhealthy food groups in the REGICOR 2000 and increases of those food groups in the REGICOR 2005 surveys. We did not reveal a constant pattern of changes, and we suppose, it was due to strong change in the proportion of participants between patterns after excluding the energy under-reporters in the REGICOR 2000.

### Effect of under-reporting on secular trends between surveys

Secular trends, changes occurred between two different samples of the same population in a certain period of time, of dietary patterns found in the present study were stable in sociodemographic and lifestyle characteristics. We found increases only in two variables: physical activity, especially in the “healthy” (19.8%) and “mixed” (11.8%) patterns, and level of education in all patterns. Lifestyle characteristics were more stable than dietary characteristics. Several changes in quantity of food consumption occurred from 2000 to 2005, such as the increase of soft drinks consumption in the “western” pattern (36.0%). This increase paralleled a considerable decline in consumption of wine (35.8%).

Energy under-reporters affected secular trends in food consumption in several food groups mostly in the “mixed”, in less proportion in the “western”, but not in the “healthy” patterns. Secular trends in the “healthy” pattern maintained the same direction and similar meaningful changes in food consumption both in all and plausible energy reporters (≥15% change in comparison with the REGICOR 2000 survey). Excluding energy under-reporters, the “healthy” pattern kept similar food consumption characteristics as the “healthy” pattern in original data set. An explanation for this finding could be healthy dietary habits of energy under-reporters [35,39,40], similar diet quality between all and energy under-reporters in the “healthy” cluster, according to the mMDS measurement, and low prevalence of energy under-reporters in the “healthy” cluster of the original data. These results confirm the theory that energy under-reporters tend to report healthier dietary habits [35,39,40].The strongest changes in secular trends were found in the “mixed” pattern, what was expected, as the energy under-reporting provoked dramatic change in the percentage of the participants in this dietary pattern in the REGICOR 2000 survey. The changes after excluding energy under-reporters were characterized mainly by increases in consumption of unhealthy food groups and decreases of healthy food groups. In the “western” cluster the effect was the same, but less food groups were affected. This was slightly different from the effect the energy under-reporters had on the “mixed” and “western” patterns within the REGICOR 2000 survey, but similar to the REGICOR 2005. Some food groups in the “mixed” and “western” dietary patterns even had different directions of secular trends between all participants and plausible energy reporters. Since the large proportion of the energy under-reporters were excluded from the “mixed” pattern, the healthy food groups consumption in this pattern decreased and unhealthy food groups increased. However, it is difficult to draw any strong conclusions, as the proportion of the participants in the “mixed” pattern of the REGICOR 2000 survey decreased dramatically. Therefore, secular trends of prevalence of participants within the “mixed” pattern substantially increased and within “healthy” and “western” patterns decreased. These results highlight an impact of energy under-reporting on time trends in nutritional surveys. Energy under-reporting influenced consumption of both healthy and unhealthy food groups in different directions, therefore, it is difficult to predict how under-reporting can influence nutritional survey data analysis. Consequently, public health investigators should pay more careful attention every time they make conclusions without taking in account energy under-reporting.

To the best of our knowledge, only one previous study in an adult population has used the cluster approach to investigate secular trends of dietary patterns [5]. The study was performed in Brazil and two dietary patterns were revealed through surveys. The patterns were stable when analysed for sociodemographic and lifestyle characteristics, and for food consumption. The diet quality index remained constant, although the timeframe for manifestation of greater changes was very short (2007–2009). The increase in soft drink consumption was similar to our findings. Two explored patterns were similar to our “healthy” and “western” patterns, but the distribution of the individuals was uneven between the patterns (86.4–90.5% vs. 9.5–12.5%, respectively). A study from Korea also used the cluster approach in secular trends analysis but it was performed in adolescents [4] The authors defined three analogous dietary patterns and impairment of dietary habits over time. In another study in adolescents, this time in the USA [3], the authors found stable patterns with principal component analysis. The patterns changed only slightly between 1998–1999 and 2003–2004. The only difference of note was the emergence of a new “fast food” pattern in boys; the patterns in girls were almost identical at both time points.

Besides cluster analysis, a priori analysis of dietary patterns has also been used to define secular trends in population dietary habits. Two independent studies in the USA analysed secular trends of dietary patterns using Heart Disease Prevention Eating Index [41] and Revised Diet Quality Index [42]. The timeframes of both surveys were long (20 and 30 years, respectively), reasonably allowing for major changes in dietary habits. Both studies revealed an overall improvement in the diet. Another study analysed secular trends for the traditional diet in Italy [43], using the Mediterranean Adequacy Index. In contrast with the USA studies, the diet of one geographic area of the Italian study sample underwent dramatic changes in all age ranges. The Mediterranean Adequacy Index decreased from 8.2–10.6 in 1967 to 2.9–6.2 in 1999. None of the mentioned studies above took into account energy under-reporting.

To look more carefully at the effect of energy underreporting on the secular trends of food consumption, we performed an additional analysis with data pooled from both surveys. The clusters from the pooled data did not differ from the clusters of the separate surveys. In one cluster solution of the plausible energy reporters, the proportion of the “mixed” cluster was dramatically decreased, as well as in the REGICOR 2000 data. In this manuscript we decided to focus on the effect of the energy under-reporters on secular trends, and pooled analysis is a good additional analysis, but it cannot fully cover the topic. Therefore, we preferred to use separate analysis.

### General characteristics of energy under-reporters

To the best of our knowledge the present study is the first to analyse dietary patterns of energy under-reporters separately from plausible energy reporters. Therefore, we briefly discuss the main features of energy under-reporters and dietary patterns associated with energy under-reporting. The characteristics of energy under-reporters in the present study were echoed in the earlier investigations. Bailey et al. [35] demonstrated that energy under-reporters had higher BMI and waist circumference, and lower education than plausible energy reporters, but unlike in our study they smoked more. Additionally, they reported lower lipids consumption, on average 400 kcal less than plausible energy reporters. Similar characteristics were found in another study [32], where energy under-reporters also had higher BMI and lower education. As in the present study, they were older and more active than plausible energy reporters. The reported dietary habits of energy under-reporters differed in comparison with all reporters in all dietary patterns, which highlights the importance of considering energy under-reporters in the analysis of dietary data in nutritional epidemiology.

Three separate dietary patterns were identified in the energy under-reporters. This demonstrates that energy under-reporters also reported different dietary habits, and not always trending toward the consumption of healthier foods. The “western” pattern, known as the least healthy pattern, was identified with a similar proportion of individuals in the dietary patterns of all participants, of plausible energy reporters and of energy under-reporters. It has been shown that healthy dietary patterns are more prevalent in energy under-reporters compared to plausible energy reporters [36,38]. Therefore, we expected the energy under-reporters to report a healthier diet than the plausible energy reporters [35,39,40]. However, energy-adjusted mMDS showed higher diet quality in energy under-reporters than in plausible energy reporters only in the “western” pattern in the REGICOR 2000 survey and in the “mixed” and “western” patterns in the REGICOR 2005 survey. On the other hand, these results are not surprising because energy under-reporters usually report lower amounts of all consumed foods along with higher proportion of intakes from foods perceived as healthy. Therefore, the diet score based on relative amounts, as in case of the mMDS, showed the reasonable values.

A limitation of the present study is the use of the FFQ, which could cause recall bias and provides an approximate amount of the consumed foods using an absolute measurement. Cluster analysis has its own weaknesses, such as arbitrary decisions made during the process of dietary patterns derivation, including number of clusters, type and standardization of variables, formation of food groups, etc. Furthermore, the Goldberg method is an indirect measure of energy misreporting, but is considered a reasonable approach in the face of the impossibility of applying a technique such as doubly labeled water in large-scale epidemiological studies to objectively measure energy underreporting. Assignation of a single PAL of 1.55 was based on the assumption of low activity levels among study participants, which results in a poor sensitivity to detect energy underreporters [44]. Additionally, the Schofield equations have been found to underestimate energy underreporters in obese individuals [45]. Therefore, we used the Mifflin equation to calculate BMR and applied individual PAL values, which improved sensitivity [44]. Finally, the results obtained in this study are population-specific and cannot be compared directly with the results in other populations.

Strengths of our study include the population-based design and the use of validated questionnaires. To our knowledge, no study has reported the results of separate cluster analysis comparing three groups: all participants, plausible energy reporters, and energy under-reporters.

This study contributes to the growing knowledge about the role of energy under-reporting in the analysis of dietary data and, particularly, in the exploration of dietary patterns defined by cluster analysis. In conclusion, energy under-reporting did not affect the structure of dietary patterns derived in 2000 and 2005, but did have an impact on the distribution of participants between dietary patterns and surveys, and influenced secular trends of food consumption and amounts of food consumed in the “mixed” and “western” dietary patterns. Analogous studies in other populations are needed to obtain a deeper understanding of the impact of energy under-reporting on the exploration of dietary data.
